# Association of allergic rhinitis with obstructive sleep apnea: A meta-analysis

**DOI:** 10.1097/MD.0000000000013783

**Published:** 2018-12-21

**Authors:** Yuan Cao, Shuang Wu, Liyu Zhang, Ying Yang, Sancheng Cao, Qiao Li

**Affiliations:** a Department of Pulmonary and Critical Care Medicine, The Second Affiliated Hospital of Medical College, Xi’an Jiaotong University; b Clinical Laboratory; c Institute of Pediatric Diseases, The Affiliated Children Hospital of Xi’an Jiaotong University, Xi’an, Shaanxi, China.

**Keywords:** allergic rhinitis, apnea hypopnea index, body mass index, Epworth Sleep Scale score, meta-analysis, obstructive sleep apnea

## Abstract

**Background:**

The co-existence of allergic rhinitis (AR) and obstructive sleep apnea (OSA) is a common phenomenon in clinical practice. AR has long been considered a risk factor for OSA. However, the relationship is not completely clear. Therefore, we conducted a meta-analysis to evaluate the prevalence of AR in sleep-disordered breathing (SDB) /OSA and their relationship.

**Methods:**

A comprehensive literature search was performed in PubMed/Medline, Google Scholar, Wiley Online Library, EMBASE, and Web of Science. Data were analyzed and pooled to estimate effect size (ES) /odds ratio (OR) with 95% confidence intervals (95%CI). Heterogeneity was quantified and evaluated by chi-squared-based Q-test and I2 test, with *P < *.05 and *I*^2^ > 50% indicating evidence of heterogeneity.

**Results:**

44 studies contained 6086 participants were included in this meta-analysis. For adults, the prevalence of AR was 22.8 (95% CI, 15.0–30.6) % in SDB and 35.2 (95% CI, 25.6–44.7) % in OSA. In children with SDB and OSA, the prevalence of AR was 40.8 (95% CI, 24.3–57.2) %, and 45.2 (95% CI, 25.4–65.0) % respectively. The odds ratios of prevalence of the SDB pediatric patients with AR was 2.12 (95%CI, 1.75, 2.57; *P < *.0001) times higher than that of non-SDB pediatric patients. There were no significant differences between OSA adults with or without AR in BMI (Body Mass Index), neck circumference, apnea hypopnea index (AHI) and epworth sleep scale score (ESS).

**Conclusion:**

The prevalence of AR in OSA/SDB is considerably high and children with SDB suffering from a higher incidence of AR than non-SDB. OSA adults accompanied with AR do not have any influences on sleep parameters.

## Abstract 2

Supplemental Digital Content is available in the text

## 1 Introduction

Obstructive sleep apnea (OSA) is the most common sleep-disordered breathing (SDB). The prevalence of OSA in healthy children and adults was as high as 1% to 5% and 3.5% to 20.4%, respectively, and it was even higher for SDB.^[1–4]^ OSA is characterized by prolonged partial upper airway obstruction and/or intermittent complete obstruction. It disrupts normal ventilation and patterns during sleep.^[1]^ Moreover, these breathing disorders may increase the risks of complications of cardiovascular, neurocognitive, and metabolic morbidities.^[5]^ Allergic diseases, allergic rhinitis (AR), asthma, and eczema are common among individuals. AR is a very common disease that affects 10% to 40% of the global population.^[6]^ With the increasing exposure to allergens and pollutants, the prevalence of AR has increased over the past few decades.^[7]^ Since it usually leads to nasal obstruction and increased upper airway resistance, AR has long been recognized as a risk factor of OSA in previous studies.^[8–10]^ Some articles have reviewed the association between AR and SDB in children. However, no meta-analysis was obtained from databases. Therefore, in view of this scenario, we conducted a meta-analysis to explore and summarize the prevalence and association between OSA and AR in order to gain a deeper insight of these 2 diseases.

## 2 Materials and methods

We performed this meta-analysis in accordance with Preferred Reporting Items for Systematic Reviews and Meta-Analyses (PRISMA) statement.^[11]^

### 2.1 Literature search

In accordance with the PRISMA guidelines, we identified relevant research articles through a systematic review of scientific databases (PubMed /Medline, Google Scholar, Wiley Online Library, Embase, and Web of Science). The MeSH and keywords used in different logical combinations and phrases were: allergic rhinitis, allergic rhino conjunctivitis, hay fever, nasal allergy, OSA, apnea, hypopnea, Epworth Sleepiness Scale (ESS), apnea-hypopnea index (AHI), SDB, sleep-associated breathing disorder, sleep-related disordered breathing, snoring. The search encompassed original research papers published by July 1, 2017 in online journals in English language.

### 2.2 Inclusion and exclusion criteria

Inclusion criterion was clinical or epidemiological studies which examined the relationship between AR and sleep disorders and reported the prevalence of AR in OSA or SDB patients. Studies were excluded from the meta-analysis if reported only the sleep quality measures other than OSA or SDB, or provided qualitative information only. Other studies that were excluded include review article, conference abstract, article not published in English, animal study, case report, article with no abstract/full text available.

### 2.3 Data extraction

The following data were extracted from each eligible study: participants’ demographic and clinical characteristics, the prevalence of AR in OSA (diagnosed with sleep studies according to guidelines),^[1,12]^ and SDB (one or more abnormal/difficulty breathing during sleep and/or gas exchange patterns during sleep including habitual snoring 3 or more times per week) patients, body mass index (BMI), neck circumference, AHI and ESS score, and other relevant information were obtained from the selected research articles of the respective studies and organized on data sheets. To ensure the quality of the meta-analysis, all eligible publications were reviewed by 2 researchers independently according to the standardized approach and later cross checked the work of each other. The final selection of a study for inclusion in the meta-analysis was reached in consensus.

### 2.4 Statistical analyses

Random effects meta-analyses were performed with STATA 12.0 (Stata Inc. Texas) to achieve overall effect sizes of the prevalence of AR in OSA and SDB patients and to achieve a summary estimate of the odds ratio of the prevalence of AR between OSA/SDB and non-OSA/SDB patients observed in the individual studies. The significance of differences in BMI, neck circumference, AHI, and ESS between OSA/SDB patients with and without AR were carried out by STATA 12.0 with under random effects model. Heterogeneity was quantified and evaluated by the chi-squared-based *Q*-test and *I*^2^ test, with *P < *.05 and *I*^2^ > 50% indicating evidence of heterogeneity.

## 3 Results

**Figure 1:**
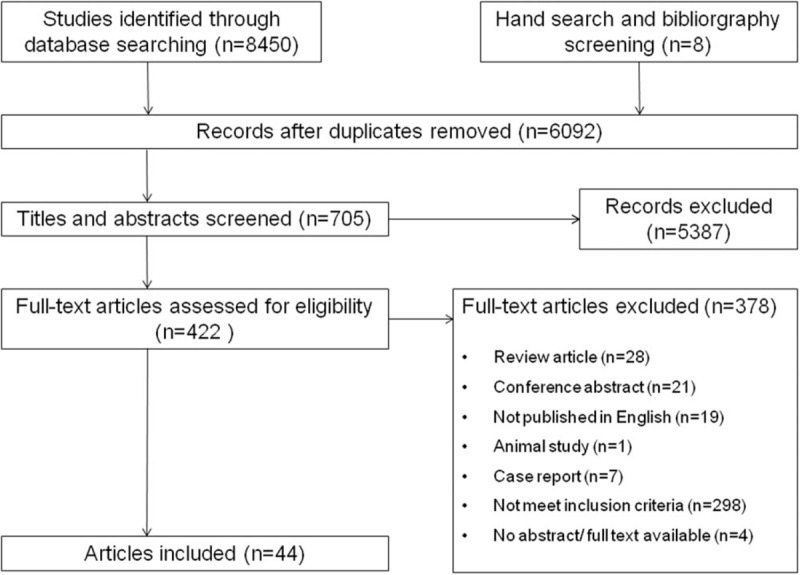
Flowchart of study screening and selection process.

Data were acquired from 44 studies ^[8,13–56]^ (6086 patients) which fulfilled the eligibility criteria (Fig. 1). Important characteristics of the included studies are presented in Table 1. Average age of adult SDB patients was 47.97 ± 4.00 years and 7.73 ± 3.34 years for SDB children. Proportion of males in this sample population was 68.55 ± 20.85% in adults and 62.09 ± 12.17% in children. In adult SDB and OSA patients, the prevalence of AR was 22.8 (95%CI, 15.0–30.6)%, and 35.2 (95%CI, 25.6–44.7)%, respectively (Fig. 2 A). In children SDB and OSA patients, the prevalence of AR was 40.8 (95%CI, 24.3–57.2)% and 45.2 (95%CI, 25.4–65.0)%, respectively (Fig. 2 B). The overall prevalence of AR (in SDB and OSA) was 41.6 (95%CI, 23.1–60.1)% in Asia, and 33.5 (95%CI, 25.3–41.6)% in other continents (Europe, Oceania, and America, Fig. S1). Pooling analysis of odds ratios observed in the children's studies showed that the prevalence of the AR was 2.12 (95%CI, 1.75, 2.57; *P*<.0001) times higher in SDB patients than in non-SDB patients (Fig. 3A). However, in adult studies, the prevalence of AR showed no significant difference between SDB/OSA patients and non-SDB/non-OSA patients (*P = *.082; *P = *.078, Fig. 3B). In adults, there was no significant difference between OSA with AR and OSA without AR in BMI (mean difference: −0.19 [95%CI, −0.45–0.07]; *P = *.149), neck circumference (mean difference: 0.16 [95%CI, −0.11–0.42]; *P* = .245), AHI (mean difference: −0.52 [95%CI, −1.79–0.74]; *P = *.416), or ESS (mean difference: 0.21 [95%CI, −0.15–0.58]; *P = *.246, Fig. S2).

**Table 1:**
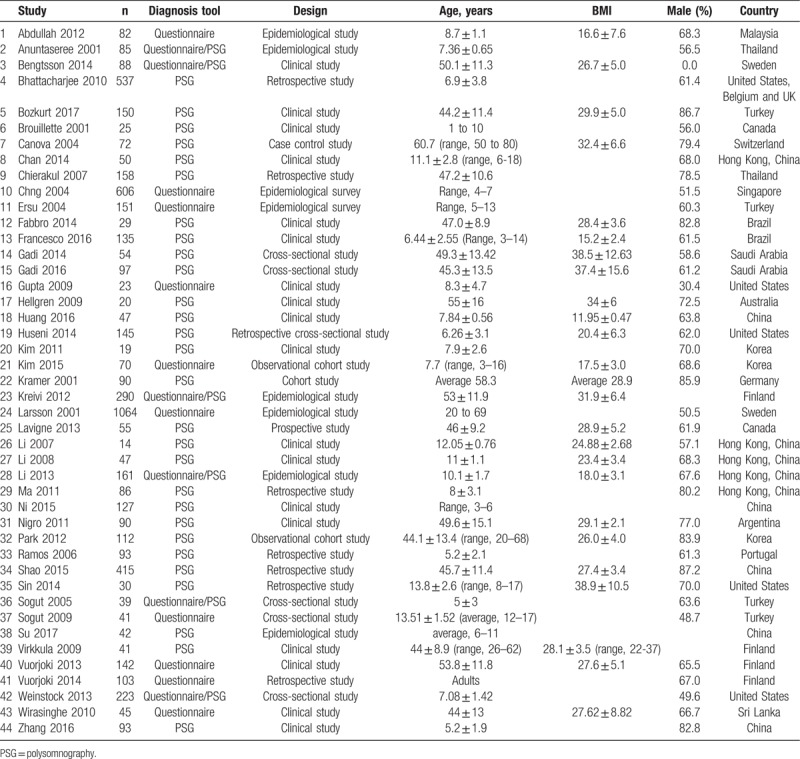
Characteristics of the included studies.

**Figure 2:**
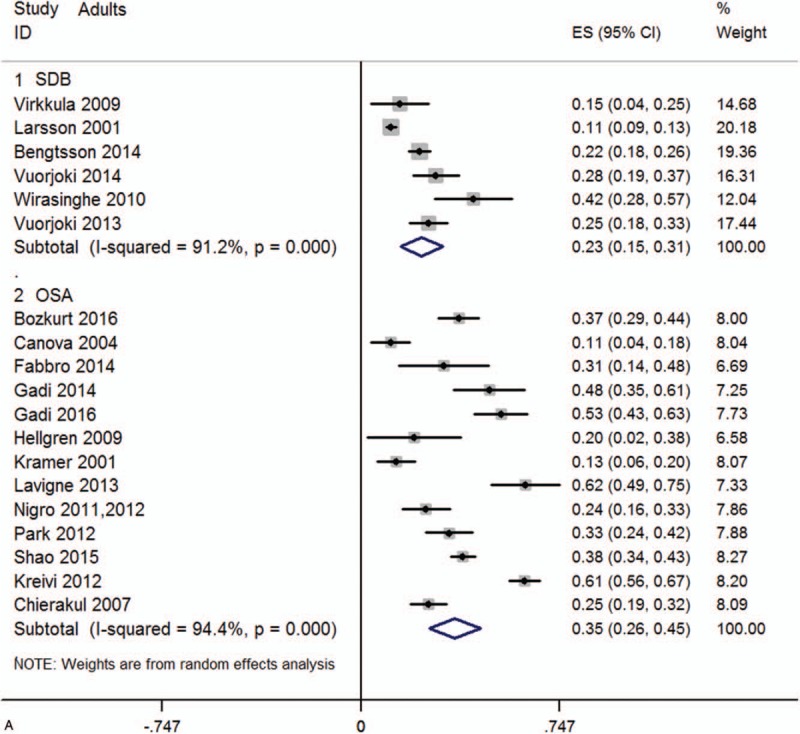
(A) Forest graph showing the percent prevalence of AR in SDB and OSA children. (B) Forest graph showing the percent prevalence of AR in SDB and OSA adults. AR= allergic rhinitis, OSA= obstructive sleep apnea, SDB= sleep-disordered breathing.

**Figure 2 (Continued):**
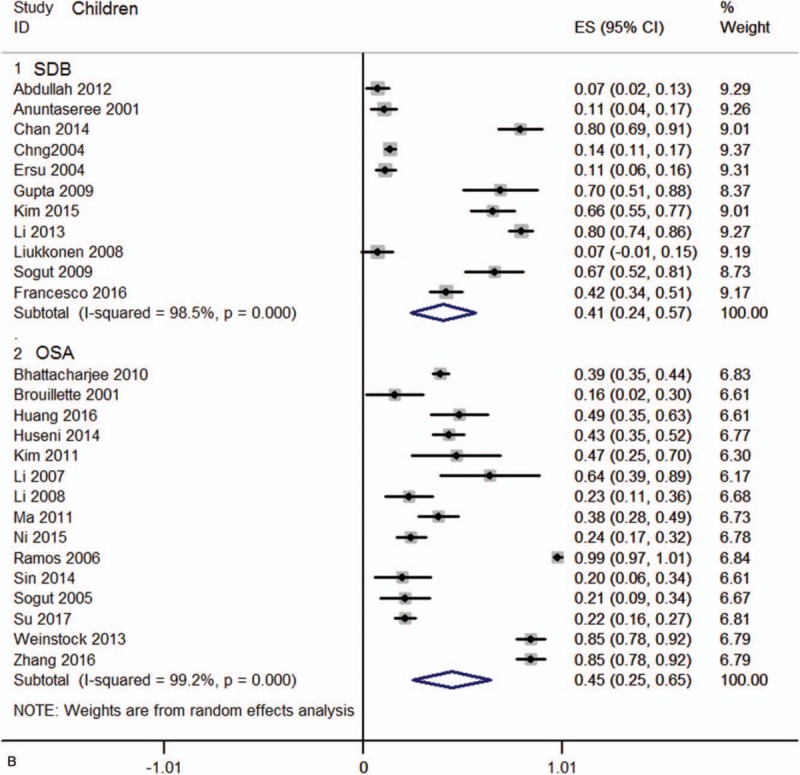
(A) Forest graph showing the percent prevalence of AR in SDB and OSA children. (B) Forest graph showing the percent prevalence of AR in SDB and OSA adults. AR= allergic rhinitis, OSA= obstructive sleep apnea, SDB= sleep-disordered breathing.

**Figure 3:**
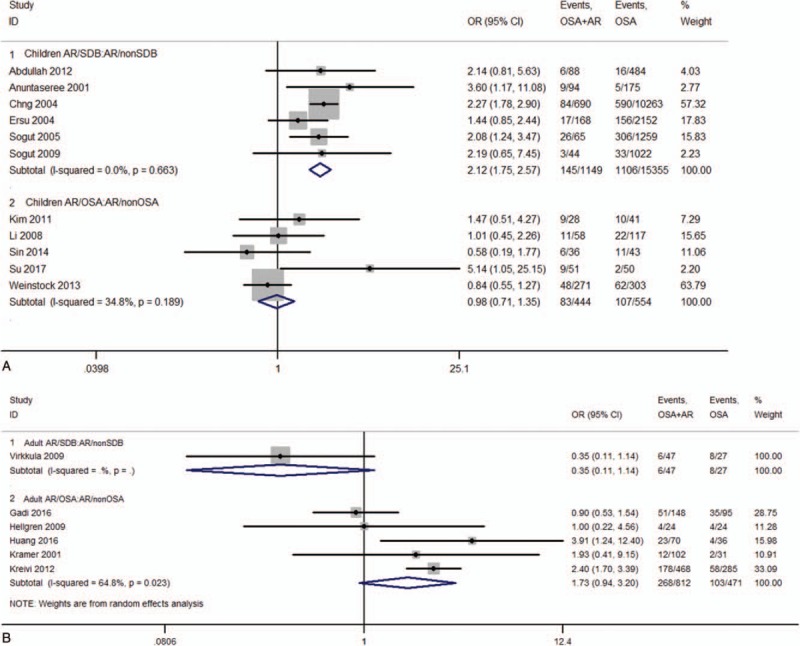
Forest graph showing the meta-analysis of odds ratios reported in individual studies with regard to the odds of prevalence of AR in SDB/OSA and non-SDB/OSA [(A) Children, (B) Adults]. AR= allergic rhinitis, OSA= obstructive sleep apnea, SDB= sleep-disordered breathing.

## 4 Discussion

Nasal obstruction had long been considered as one of the leading risk facts for the upper airway obstruction during the sleep. Further, some clinical studies had found that patients with nasal congestion caused by AR were more susceptible to disturbed sleep.^[57–59]^ Rhinorrhea, nasal blockage, or congestion always led to stuffy nose, which were the most complained symptoms of AR patients.^[60]^ The nasal obstruction may gradually increase, resulting in daytime fatigue, sleepiness, and performance decrements. In addition, symptoms brought by AR such as apnea and snoring were also considered to be risk factors for sleep-disordered breathing events and contributed to the development of OSA.^[61]^ In addition, some studies had demonstrated that several chemical mediators and inflammatory cytokines play interaction roles between AR and OSA, including histamine, cysteinyl leukotrienes (cysLTs), interleukin-1β(IL-1β), and interleukin-1 (IL-4) and so on.^[58,62]^ Given the impact of AR to OSA, it seemed logical to investigate their relationship which may help understand overlapped subjects.^[62]^

In this meta-analysis, we reported that the prevalence of children diagnosed with AR is 2.12 times higher in SDB patients than that of non-SDB patients. However, we did not find this tendency in adults, due to immaturity of immune system in children and the studies’ bias. AR was one of the most common chronic diseases in children. Children's immune system developed around the age of 2-year old. During this time, they were more likely to acquire Type I hypersensitivity which reflecting more T helper 2 lymphocyte (Th2) and consequently immunoglobulin E (IgE) driven response to allergen exposure.^[63]^ Adenotonsillar hypertrophy (AH) was a common comorbidity of pediatric AR, it was reported that 92.6% of AR children also suffered AH .^[61]^ Furthermore, AH is the main cause of OSAS for children aged 3 to 6, yet adults patients were often caused by obesity.^[64]^ This meta-analysis showed no significant difference in the prevalence of AR in OSA and non-OSA patients (children and adults). This would lead to widely underdiagnosis of OSA in group of patients in clinical practice.^[65,66]^ Although weight and neck circumference had been shown to be good predictors for OSA in epidemiologic studies, our study suggested that there was not enough evidence that OSA adults patients coexist AR were related to individuals’ BMI, neck circumference, ESS, and AHI. Additionally, Francesco and Alvarez^[24]^ reported that AR is not an aggravating factor regarding the severity of AHI in children. The relationship between the OSA and AR is remaining a long-standing controversy. In traditional view, AR was considered as a potential risk factor for OSA. However, Kramer et al^[33]^ revealed that AR did not influence sleeping parameters of OSA. They found no statistically significant difference in sleeping behavior or polysomnography (PSG) parameters between AR and non-AR patients. Recent studies had also revealed AR only had effect on symptoms, but did not affect PSG results for OSA patients nor belong to risk factors for OSA.^[15,17]^ Similarly, a demographic study conducted in OSA children reported that allergic rhinitis did not contribute to sleep disordered breathing in Australian.^[67]^ Intranasal corticosteroids (INCS) were generally considered as the most effective agent in relieving nasal symptoms of allergic rhinitis. Therefore, it was believed that the effectiveness of INCS in relieving nasal congestion may have a positive effect on SDB. A randomized, controlled trial of OSA children aged 6 to 18 years old demonstrated that intranasal mometasone furoate effectively improved obstructive apnea hypopnea index and oxygen desaturation index.^[20]^ Beyond that, Lavigne et al^[36]^ found that INCS not only reduced upper airway inflammation but also improved OSA morbidity in patients with concomitant AR. A meta-analysis showed that patients receiving INCS had a better effect on decreasing the AHI; however, with limited evidences.^[68]^ In general, continuous positive airway pressure (CPAP) was the preferred therapy for OSA, whereas, INCS did not alleviate nasal symptoms during CPAP treatment in OSA patients.^[69]^ Hence, further research should be performed on INCS in these 2 entities.

In conclusion, patients with AR were more likely to become habitual snorers, and had increasing risk of SDB and the SDB children suffered from a higher incidence of AR. Physicians may need a comprehensive understanding of the overlapping disorders before making a reasonable therapeutic strategy.

## 5 Conclusion

This meta-analysis revealed that the prevalence of AR in adult SDB/OSA patients was 23%, and 35%, and in children SDB/OSA patients 41%, and 45%, respectively. The odds of having AR were 2.12 times higher in SDB than that of non-SDB children patients (significantly). However, there was no significant difference between OSA patients and those who suffered AR and OSA in neck circumference simultaneously BMI, AHI, or ESS. Whether AR is a risk event of OSA need further consideration, meanwhile, patients with SDB should be cautiously focused especially accompanied with nasal symptoms.

## Author contributions

**Conceptualization:** Yuan Cao, Qiao Li.

**Data curation:** Yuan Cao, Liyu Zhang.

**Formal analysis:** Yuan Cao, Ying Yang.

**Funding acquisition:** Liyu Zhang, Sancheng Cao.

**Writing – original draft:** Shuang Wu.

**Writing – review & editing:** Qiao Li.

Qiao Li: 0000-0002-2575-7005.

QIAO LI orcid: 0000-0002-2575-7005.

## Supplementary Material

Supplemental Digital Content
