# Supplementary material for: Association of allergic rhinitis with obstructive sleep apnea: A meta-analysis
Source: Medicine (Baltimore). 2018 Dec 21;97(51):e13783. doi: 10.1097/MD.0000000000013783 (PMC6319794; doi:10.1097/MD.0000000000013783)
Supplement: Supplemental Digital Content [file medi-97-e13783-s001.pdf]

Fig. S1 Forest graph showing the overall prevalence of AR in Asia, and other continents (Europe, Oceania, and America.

Fig. S2 Forest graph showing the mean difference between OSA adults with AR and without AR in BMI, neck circumference, AHI an

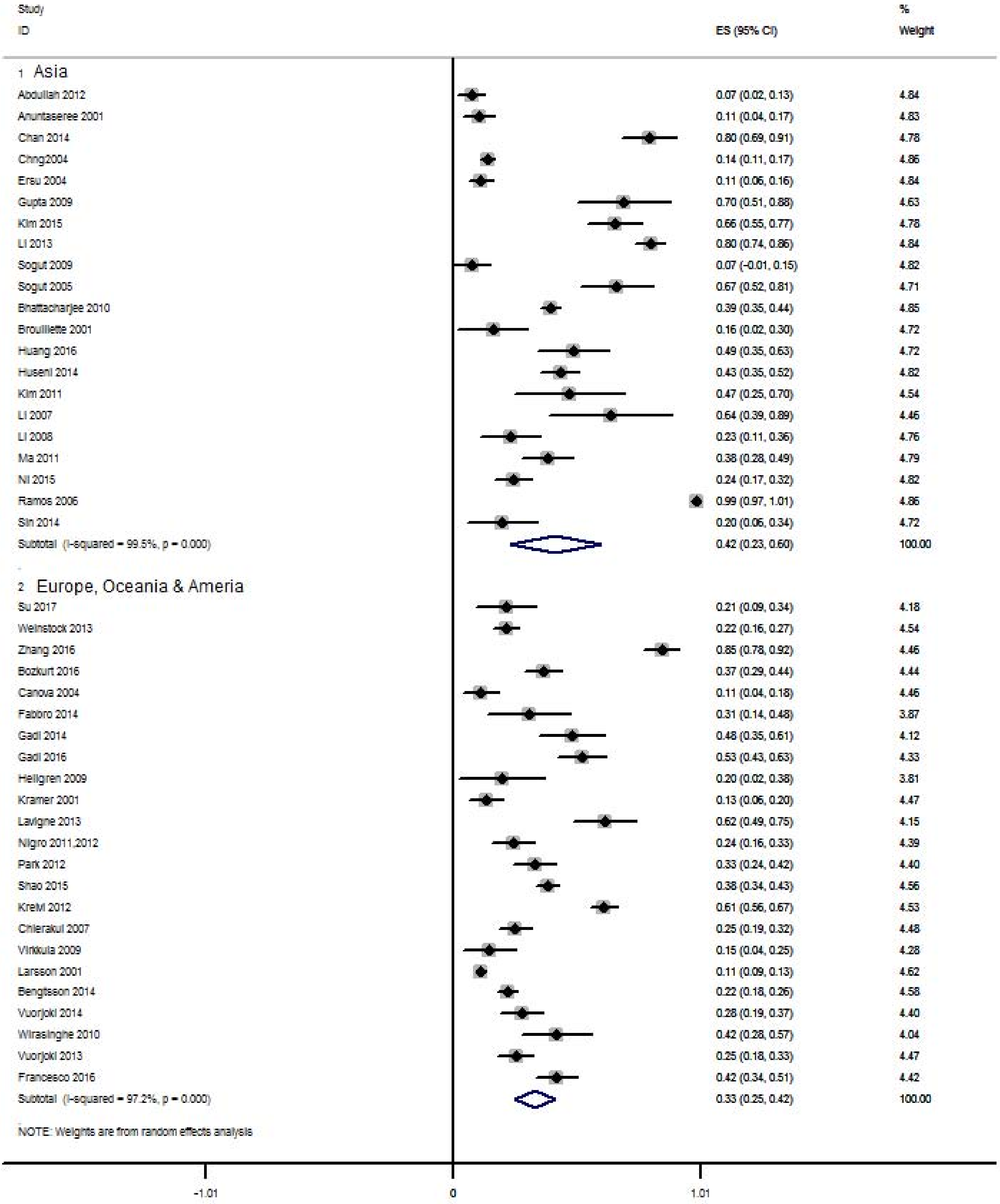
Study
ID
ES (95% CI)
%
Weight
1 Asia
Abdullah 2012 0.07 (0.02, 0.13) 4.84
Anuntaseree 2001 0.11 (0.04, 0.17) 4.83
Chan 2014 0.80 (0.69, 0.91) 4.78
Chng2004 0.14 (0.11, 0.17) 4.86
Ersu 2004 0.11 (0.06, 0.16) 4.84
Gupta 2009 0.70 (0.51, 0.88) 4.63
Kim 2015 0.66 (0.55, 0.77) 4.78
Li 2013 0.80 (0.74, 0.86) 4.84
Sogut 2009 0.07 (-0.01, 0.15) 4.82
Sogut 2005 0.67 (0.52, 0.81) 4.71
Bhattacharjee 2010 0.39 (0.35, 0.44) 4.85
Brouilliette 2001 0.16 (0.02, 0.30) 4.72
Huang 2016 0.49 (0.35, 0.63) 4.72
Huseni 2014 0.43 (0.35, 0.52) 4.82
Kim 2011 0.47 (0.25, 0.70) 4.54
Li 2007 0.64 (0.39, 0.89) 4.46
Li 2008 0.23 (0.11, 0.36) 4.76
Ma 2011 0.38 (0.28, 0.49) 4.79
Ni 2015 0.24 (0.17, 0.32) 4.82
Ramos 2006 0.99 (0.97, 1.01) 4.86
Sin 2014 0.20 (0.06, 0.34) 4.72
Subtotal (I-squared = 99.5%, p = 0.000) 0.42 (0.23, 0.60) 100.00
2 Europe, Oceania & Ameria
Su 2017 0.21 (0.09, 0.34) 4.18
Weinstock 2013 0.22 (0.16, 0.27) 4.54
Zhang 2016 0.85 (0.78, 0.92) 4.46
Bozkurt 2016 0.37 (0.29, 0.44) 4.44
Canova 2004 0.11 (0.04, 0.18) 4.46
Fabbro 2014 0.31 (0.14, 0.48) 3.87
Gadi 2014 0.48 (0.35, 0.61) 4.12
Gadi 2016 0.53 (0.43, 0.63) 4.33
Hellgren 2009 0.20 (0.02, 0.38) 3.81
Kramer 2001 0.13 (0.06, 0.20) 4.47
Lavigne 2013 0.62 (0.49, 0.75) 4.15
Nigro 2011,2012 0.24 (0.16, 0.33) 4.39
Park 2012 0.33 (0.24, 0.42) 4.40
Shao 2015 0.38 (0.34, 0.43) 4.56
Kreivi 2012 0.61 (0.56, 0.67) 4.53
Chierakul 2007 0.25 (0.19, 0.32) 4.48
Virkkula 2009 0.15 (0.04, 0.25) 4.28
Larsson 2001 0.11 (0.09, 0.13) 4.62
Bengtsson 2014 0.22 (0.18, 0.26) 4.58
Vuorjoki 2014 0.28 (0.19, 0.37) 4.40
Wirasinghe 2010 0.42 (0.28, 0.57) 4.04
Vuorjoki 2013 0.25 (0.18, 0.33) 4.47
Francesco 2016 0.42 (0.34, 0.51) 4.42
Subtotal (I-squared = 97.2%, p = 0.000) 0.33 (0.25, 0.42) 100.00
NOTE: Weights are from random effects analysis
-1.01
0
1.01

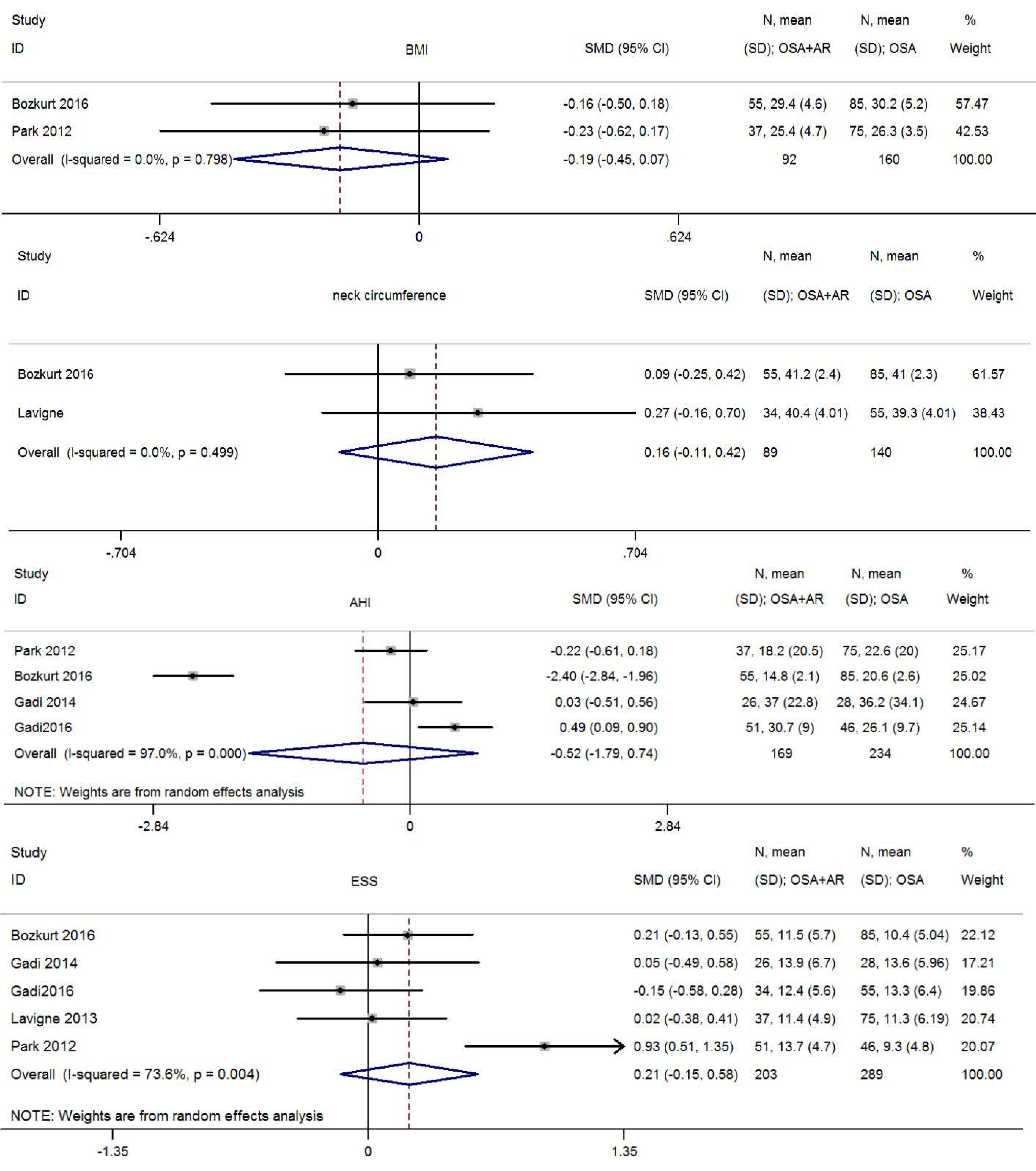
Study
ID
BMI
SMD (95% CI)
N, mean (SD); OSA+AR
N, mean (SD); OSA
% Weight
Bozkurt 2016
-0.16 (-0.50, 0.18)
55, 29.4 (4.6)
85, 30.2 (5.2)
57.47
Park 2012
-0.23 (-0.62, 0.17)
37, 25.4 (4.7)
75, 26.3 (3.5)
42.53
Overall (I-squared = 0.0%, p = 0.798)
-0.19 (-0.45, 0.07)
92
160
100.00
-.624
0
.624
Study
ID
neck circumference
SMD (95% CI)
N, mean (SD); OSA+AR
N, mean (SD); OSA
% Weight
Bozkurt 2016
0.09 (-0.25, 0.42)
55, 41.2 (2.4)
85, 41 (2.3)
61.57
Lavigne
0.27 (-0.16, 0.70)
34, 40.4 (4.01)
55, 39.3 (4.01)
38.43
Overall (I-squared = 0.0%, p = 0.499)
0.16 (-0.11, 0.42)
89
140
100.00
-.704
0
.704
Study
ID
AHI
SMD (95% CI)
N, mean (SD); OSA+AR
N, mean (SD); OSA
% Weight
Park 2012
-0.22 (-0.61, 0.18)
37, 18.2 (20.5)
75, 22.6 (20)
25.17
Bozkurt 2016
-2.40 (-2.84, -1.96)
55, 14.8 (2.1)
85, 20.6 (2.6)
25.02
Gadi 2014
0.03 (-0.51, 0.56)
26, 37 (22.8)
28, 36.2 (34.1)
24.67
Gadi2016
0.49 (0.09, 0.90)
51, 30.7 (9)
46, 26.1 (9.7)
25.14
Overall (I-squared = 97.0%, p = 0.000)
-0.52 (-1.79, 0.74)
169
234
100.00
NOTE: Weights are from random effects analysis
-2.84
0
2.84
Study
ID
ESS
SMD (95% CI)
N, mean (SD); OSA+AR
N, mean (SD); OSA
% Weight
Bozkurt 2016
0.21 (-0.13, 0.55)
55, 11.5 (5.7)
85, 10.4 (5.04)
22.12
Gadi 2014
0.05 (-0.49, 0.58)
26, 13.9 (6.7)
28, 13.6 (5.96)
17.21
Gadi2016
-0.15 (-0.58, 0.28)
34, 12.4 (5.6)
55, 13.3 (6.4)
19.86
Lavigne 2013
0.02 (-0.38, 0.41)
37, 11.4 (4.9)
75, 11.3 (6.19)
20.74
Park 2012
0.93 (0.51, 1.35)
51, 13.7 (4.7)
46, 9.3 (4.8)
20.07
Overall (I-squared = 73.6%, p = 0.004)
0.21 (-0.15, 0.58)
203
289
100.00
NOTE: Weights are from random effects analysis
-1.35
0
1.35
